# Exploring and translating novel approaches targeting the choroid plexus for the treatment of hydrocephalus: a literature review

**DOI:** 10.1186/s12987-026-00779-5

**Published:** 2026-07-31

**Authors:** Olivier J. J. Sluijters, John R. Pooley, William G. B. Singleton

**Affiliations:** 1 Department of Neurosurgery, North Bristol NHS Trust, Bristol, BS10 5NB UK; 2 Translational Health Sciences, Bristol Medical School, University of Bristol, Bristol, BS8 1TD UK; 3 Department of Paediatric Neurosurgery, Bristol Royal Hospital for Children, Bristol, BS2 8BJ UK; 4 Living Systems Institute, University of Exeter, Exeter, EX4 4PY UK

**Keywords:** Hydrocephalus, Choroid plexus, Paediatric neurosurgery, Post-haemorrhagic hydrocephalus, Hypersecretion, Inflammatory response, Endoscopic coagulation of choroid plexus, CSF diversion

## Abstract

Hydrocephalus is common with a mixed phenotype and aetiology. Its treatment is predominantly surgical, with temporary or permanent cerebrospinal fluid (CSF) diversion, which does not address the underlying disease mechanism. The choroid plexus (ChP), a secretory epithelium found in all four ventricles of the brain, produces most CSF within the central nervous system and has been investigated extensively over the past century to understand its function and potential role as a therapeutic target. Past attempts at either medically or surgically controlling its rate of secretion have not significantly altered our approach to the treatment of hydrocephalus, with CSF diversion remaining the main intervention. Rodent models of post-haemorrhagic hydrocephalus (PHH) have advanced our understanding of choroid plexus function in health and disease. Pre-clinical experiments have demonstrated a hypersecretory response in the ChP that may contribute to PHH. Targeting the choroid plexus directly may therefore present a novel therapeutic method. These findings have led to a significant increase in pre-clinical studies exploring this hypersecretory response and how to modulate it. Translating these promising results to clinical practice will rely on the development of large animal hydrocephalus models, which thus far has been limited. This literature review discusses the recent advances in targeting the ChP as a treatment for hydrocephalus, both surgically and non-surgically, and the current barriers to further advancement of this approach.

## Background

CSF, which Cushing describes as ‘the peculiar watery medium which bathes the central nervous system’ in his lectures on the third circulation [1], plays a crucial role in the nervous system; aiding its development, providing protection, buoyancy, homeostasis of the ionic environment, nutrient supply and a pathway for waste clearance [2, 3]. It is predominantly produced by the ChP, with extra-choroidal contributions from the ependyma and blood-brain barrier (BBB) suggested [4, 5]. It circulates the central nervous system through ventricular, cisternal, subarachnoid and glymphatic pathways [6, 7], propelled by a combination of hydrostatic pressure, arterial pulsations, respiratory patterns and beating of cilia. Its absorption takes place in arachnoid granulations, nerve sheaths, dural lymphatics [8] and other regions [9]. The production, circulation and absorption of CSF is a rapidly evolving field of research, and traditional hypotheses are continuously challenged [10].

An abnormal accumulation of CSF in the ventricles leads to *hydrocephalus*, however the underlying pathophysiology of the processes that lead to this remain unclear. It can be defined as ‘a clinical and neuroradiographic diagnosis characterised by an abnormal accumulation of CSF which can occur in conjunction with, or in absence of, changes to intracranial pressure’ [11]. The wide range that this definition covers highlights how variable the clinical presentation of a hydrocephalic patient can be, ranging from the acutely comatose patient to a subtle cognitive decline. Originally subdivided into being caused by overproduction of CSF, a blockage of its circulation or an impaired re-absorption of CSF, it is now known that these processes frequently co-exist.

Hydrocephalus is a highly prevalent condition, affecting patients of all ages [12–14]. There are an estimated 400,000 new cases of paediatric hydrocephalus per year worldwide and it can develop as a primary pathology or secondary to an insult. The morbidity, mortality and economic cost associated with it is significant [15, 16], as emphasised by those trialling novel treatments [17].

Many non-surgical therapies have been investigated and trialled over the past century [18], however the mainstay of hydrocephalus treatment remains surgical, as medical treatment is often temporary and limited by significant side-effects [18]. Surgical treatment consists of initial temporary CSF diversion (via lumbar puncture, lumbar or external ventricular drain), permanent CSF diversion (via ventriculoperitoneal or ventriculoatrial shunt), and endoscopic techniques, such as endoscopic third ventriculostomies. An extensive array of different shunt valves, siphon devices and pressure monitors [19] is available to optimise the treatment of these patients and reduce the risks of complications (i.e. hardware failure, infection). The BASICS trial has demonstrated the efficacy of antibiotic-impregnated catheters in reducing the incidence of shunt infections requiring revision [20].

The inherently imperfect nature of surgical CSF diversion, which does not necessarily address the root cause of hydrocephalus, means that patients are frequently plagued by complications, such as hardware failure, infections and lifelong follow-up requirements [16, 21]. Endoscopic approaches are also troubled by complications, with higher early, though reduced late-failure rates [22]. Because of this, there is a drive to explore more permanent therapeutic approaches that avoid these issues.

Some of the promising advances in this field have targeted the ChP directly. Considering recent evidence that, unlike previously thought, the ChP demonstrates a hypersecretory response in post-haemorrhagic and post-infectious hydrocephalus [23], there is a growing interest in targeting it directly, with a view to reduce the need for permanent CSF diversion. This focused literature review will discuss the latest surgical and non-surgical advances in targeting the ChP for the treatment of hydrocephalus and highlight areas of novel research with translational potential.

## The choroid plexus

### Structure and development

The ChP is a secretory epithelium that is found on the floor of both lateral ventricles and the roof of both the 3rd and 4th ventricle. Within the lateral ventricles, the ChP follows and adheres to the C-shaped choroid fissure and is predominantly found in the body of the ventricle and the temporal horns. Small ridges called tenia, a continuation of the ependyma, attach the plexus to the thalamus and fornix. The epithelium is continuous with that of the ChP in the 3rd ventricle at the foramina of Monro, where it arises from the roof of the third ventricle, from the lower part of the tela choroidea. In the 4th ventricle, the ChP consists of two strands, which are divided into medial and lateral segments, and extend to the foramina of Luschka and Magendie [24]. The arterial supply is from the anterior and posterior choroidal arteries to the lateral plexuses, and the posterior cerebral and posterior inferior cerebellar artery to the 3rd and 4th ventricular plexuses respectively [25].

During foetal development, the ChP first appears in the fourth ventricle, during the 9th week of gestation after closure of the neural tube. Subsequently the third ventricle and lateral ventricle plexuses develop. These develop from the neuroepithelium and surrounding mesenchyme in the lateral, third and fourth ventricles. CSF is initially trapped amniotic fluid, and is gradually replaced with embryonic CSF, which is secreted by the surrounding neuroepithelium [26]. Initial ventricular expansion during embryonic development occurs before the ChP appears [27], so the role of CSF production is likely initially carried out by the neuroepithelium and later taken over by the choroid plexus epithelium.

On a cellular level, the ChP differs from the closely related ependymal lining of the ventricular wall. The ventricular ependyma is lined with ciliated columnar cells and lacks tight junctions, forming a non-restrictive barrier that allows relatively unrestricted passage of solutes and water [28]. Contrary to this, the ChP consists of a single layer of cuboidal epithelial cells [29] which are tightly interlinked with a range of junctional proteins, such as claudin 1, 2, 11, occludin and ZO-1 [30, 31]. This layer of epithelium is supported by a basement membrane and a rich network of fenestrated capillaries, with surrounding stromal cells and fibroblasts [29, 32]. These cellular layers combined form what is named the blood-CSF barrier (BCSFB) [33]. Choroid plexus epithelial cells display multiple features that align with their function of CSF production. These include microvilli, a basolateral border with interdigitations, high density of mitochondria [34], high expression of sodium-potassium ATPases (N-KA) [35] and membrane transport proteins [36]. The surface area of the ChP is calculated to be between 2 and 5 m^2^, which is approximately half of the entire BBB surface (10m^2^) [37]. The described anatomical, macroscopic and microscopic features of the ChP support its function as a highly active secretory unit.

### CSF production in the choroid plexus

CSF production is estimated at around 500 ml/day in the average adult, with 80–90% produced by the ChP [5, 38], the remainder coming from ependyma and brain interstitial fluid. The ChP serves multiple other functions within the CNS, such as a close control of extracellular ion homeostasis [3]. Whilst there is ongoing debate on this matter, there is a large body of evidence supporting the important role of the ChP in CSF production [33, 39], which is summarised below.

Suggested by landmark figures such as Willis, Luschka, Faivre and Frazier [40], the first experimental evidence for CSF production by the ChP was published in the early 1900s by Dandy. He demonstrated that by blocking the foramina of Monro, the lateral ventricles expand, but that if the ChP was resected from one or both ventricles, this would not occur in the respective ventricle [41].

In the 1960s, Rougemont demonstrated that filter paper rapidly saturates when pressed against exposed choroid plexus epithelium (ChPE), as opposed to the ventricular wall or pial surface, where it stayed relatively dry. He also developed a technique for directly sampling fluid from the ChP for biochemical analysis, demonstrating its similarity to fluid in the cisterna magna [42].

Welch was able to measure and calculate the rate of CSF production from rabbit ChP, by calculating blood volume lost as fluid between arterial and venous blood. They also demonstrated a decrease in production with topical application of ouabain, a cardiac glycoside [43]. Ames demonstrated similar results with topical administration of acetazolamide, a carbonic anhydrase inhibitor, and assessed responses to changes in arterial CO_2_ content by directly measuring fluid produced from the ChP with a pipette [44]. Pappenheimer developed a technique called ventriculo-cisternal perfusion, which allowed the measurement of changes in CSF volume, production and composition [45]. Whilst not a direct measure of ChP function, this technique provided much needed insight into general CSF hydrodynamics, including pressures, production and absorption rates.

The next significant step forward was by Wright et al. in the 1970s, who were able to mount bullfrog ChP in an Ussing chamber to more directly assess transepithelial transport and secretion rates. This led to an increased understanding of active transcellular transport, epithelial resistance and other characteristics of the epithelium [33, 46–48]. Subsequent studies with labelled cations demonstrated active secretion of sodium and chloride by the ChP, hinting at the underlying mechanisms and allowing evaluation of drugs, such as acetazolamide [49].

There have been opposing views that argue against a dominant role of the ChP in CSF production, such as Milhorat et al., who resected the lateral ventricle choroid plexuses in rhesus monkeys and noted only a 30–40% reduction in CSF production, as well as noting a possible absorptive role in his clinical observations [50, 51]. Others have, based on the above evidence, argued against a central role of the ChP, and instead suggest that CSF is continuously produced and absorbed throughout the central nervous system [52]. It is beyond the scope of this paper to detail such discussions, but several reviews [5] have addressed these specific concerns and provided counterarguments [53], such as the presence and contribution of the plexus in the 3rd and 4th ventricle.

### Molecular basis of CSF production by choroid plexus epithelium

Damkier, Hladky, Spector and MacAulay have produced comprehensive and detailed reviews on the experimental evidence basis for our current understanding of CSF secretion by the ChP [4, 5, 33, 37]. The key aspects are summarised below.

The ChPE is capable of transporting fluid independent of and even against an osmotic gradient. The underlying mechanism for this paradoxical fluid-transport remains unknown, with numerous theories being explored. These include a standing gradient in the lateral intercellular spaces, fluid transport through tight-junctions between choroid plexus epithelial cells (ChPECs), and direct transport of water molecules through transporter molecules (discussed in section ‘The inflammation pathway: TLR4-SPAK-pNKCC1’). All these theories have experimental and mathematical analyses that argue for and against [5, 33, 39], with no clear explanation so far as to how the ChPE can achieve this fluid transport.

**Fig. 1:**
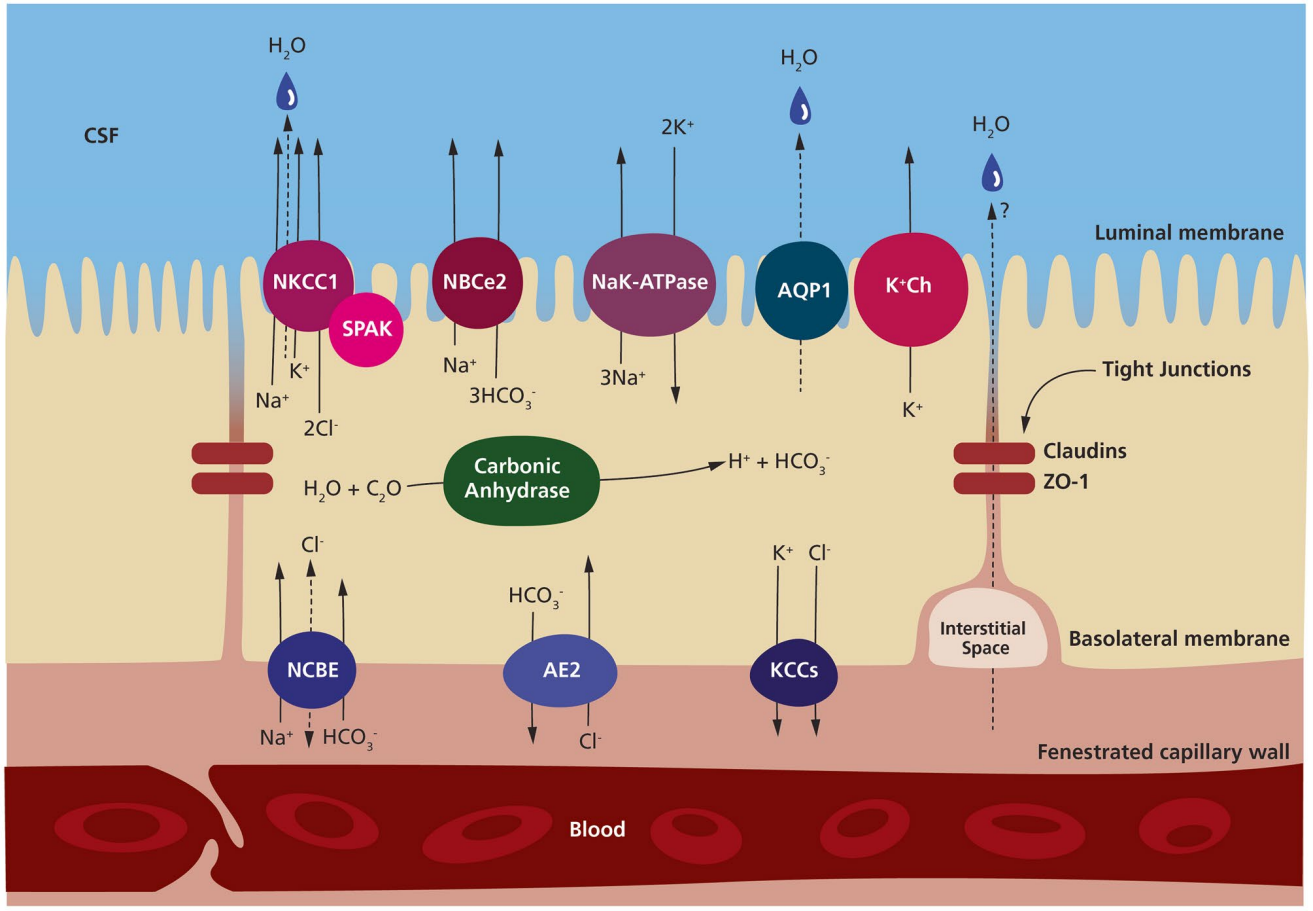
Overview of key structural and transporter molecules in the choroid plexus epithelium with the associated direction of ion and water movement. The luminal surface of the choroid plexus epithelial cell has microvilli to expand its surface area. The junctions between cells are formed by junctional proteins, such as claudins and ZO-1. There are pockets of interstitial space on the basolateral side, where the basement membrane and fenestrated capillary wall forms the remainder of the blood – CSF barrier. The main transport molecules on the luminal side are cation-chloride co-transporters (NKCC1), sodium-potassium ATPase (NaK ATPase), aquaporin 1 channels (AQP1), sodium-bicarbonate co-transporter (NBCe2) and potassium channels (K + Ch). SPAK (STE20/SPS1-related Proline-Alanine-rich Kinase) is closely associated with NKCC1 and phosphorylates the transporter, promoting its activity. On the basolateral side, the main transport molecules are sodium driven chloride-bicarbonate exchangers (NCBE), anion exchangers (AE2) and other cation cotransporters (KCCs). Carbonic anhydrase is found within the cytoplasm and catalyses the conversion of H2O and CO2 into HCO3- and H+, which is a key step in facilitating ion fluxes and fluid transport across the epithelium. Various experimental studies with knockout rodent models, topically and systemically applied inhibitors have demonstrated the existence of these molecules and their impact on CSF production, although the nature of their interactions remains unclear

Experimental evidence, using knockout rodent models, topically and systemically applied inhibitors and conduction studies, supports a role for the following channels on the apical membrane of ChPECs, which are summarised in Fig. 1: Cation-chloride co-transporters (NKCC1), Na/K-ATPase (which is uniquely located on the luminal side), aquaporin channels (AQP1), Sodium-bicarbonate co-transporters (NBCe2) and several potassium channels (KCh). On the basolateral side, there is evidence for sodium driven chloride-bicarbonate exchangers (NCBE), anion exchangers (AE2) and other cation cotransporters (KCCs). There is also an important role for carbonic anhydrase (CA), of which several isoforms are present intracellularly, the target of the only widely used medical treatment to reduce CSF production, acetazolamide.

## Surgical approaches to the choroid plexus

Conceptually the most straightforward approach to targeting the ChP is to surgically resect or damage it, reducing its capacity to produce CSF. We will discuss the evidence supporting these direct approaches, as well the evidence for surgical approaches to CSF diversion, with a view to limit the degree of blood products that the ChP is exposed to, thus preventing or minimising subsequent inflammatory reactions and potentially permanent changes.

### Endoscopic choroid plexus coagulation

The main barrier to successfully resecting the ChP in the early 1900s was the high mortality rate associated with open approaches, but since L’Espinasse, a urologist, first demonstrated an endoscopic method to coagulate the ChP in 1910, its technique has been advanced by Putnam [54] and Scarff [55]. Multiple series and trials have been done to investigate its effectiveness.

In 1986 Griffith and Syman published a series of 70 cases demonstrating that choroid plexus coagulation (CPC) led to 30–49% shunt-independence [56]. This work led to Griffith and Jamjoom trialling a ventricular perfusion protocol combined with CPC [57]. This involved two ventricular catheters and 48–72 h of constant infusion and drainage of artificial CSF, to lower the blood and protein content of the CSF. Their series of 25 patients had a 52% shunt-independence rate. Their reasoning for this protocol was a belief that the breakdown products from coagulating the ChP may affect subsequent absorption of CSF and lead to hydrocephalus. The long-term outcomes for these patients (mean follow-up of 10.5 years) demonstrate a 35% shunt-independence rate, with reduced rates of shunt-revisions in the remainder and higher independence rates in cases with slowly progressing hydrocephalus [58].

CPC has had a resurgence of use since the early 2000s, especially in conjunction with endoscopic third ventriculostomies (ETV) – it was felt to be a helpful adjunct to avoid placing a shunt in patients, especially when regular follow-up was not possible. Multiple series have been published, with Warf carrying out the majority, comparing ETV and ETV combined with CPC, demonstrating the potential benefit of CPC [59–63]. A systematic review of 16 studies in 2022 showed a significant reduction in failure rates (i.e. need to place a shunt) in the ETV + CPC group compared to ETV alone [64]. It is evident from the varying rates across studies that patient selection, surgical technique and healthcare setting have a significant impact. The analysis revealed that failure rates were higher in high-income countries compared with LMIC, which may be attributable to different underlying disease processes. Similar results are reported by an earlier meta-analysis of 5 studies, that did not find an overall benefit of ETV + CPC compared to ETV, but a subgroup analysis of African cohorts did [65].

In May 2023 a cohort study by Warf et al. of 348 children, demonstrated that regardless of aetiology, ETV + CPC results in higher success rates than the ETV success score predicts, reaffirming the added value of CPC [66]. The primary aim of this study was to compare endoscopic treatment with shunt placement.

A study of patients undergoing functional hemispherectomies for intractable epilepsy, with randomisation to additional (open) choroid plexus coagulation demonstrated that the shunt requirement was significantly lower in the CPC group (7.7% in CPC group vs. 28.7% in no CPC group) [67]. Similarly promising results were achieved in a small series of patients with hydranencephaly, where choroid plexectomy led to lower rates of CSF diversion [68].

### Choroid plexus destruction by other means

Other methods have been trialled to reduce CSF production from the ChP by destroying its tissue, such as gold, rhenium and technetium [69–73], which were initially promising but proved unsuccessful in animal models of kaolin-induced hydrocephalus.

Stereotactic radiosurgery is used to target neoplasms of the ChP, both primary and metastatic, and work has been done detailing the dose requirements to achieve sustained cell death in normal ChP tissue [74]. There are no published works yet trialling this with the primary aim of treating hydrocephalus.

Embolisation of the distal anterior choroidal arteries has been described in a case report of a child with diffuse villous hyperplasia of ChP, whose CSF production remained at 800-1000 ml/day despite CPC and developed ascites after a VP shunt [75]. In other case reports, CPC and medical therapy have been adequate to manage the excessive CSF production [76], but in this case, neuroradiological intervention was planned. CSF production declined to 200-300 ml/day after bilateral embolization of the anterior choroidal artery (at the plexal point, to avoid supply to adjacent critical structures), though this did lead to a silent posteromedial thalamic infarct. There have been no other reports of this approach in the literature, except in pre-operative settings for the resection of ChP tumours [77, 78], or treatment of vascular malformations, which highlight the variable anatomy of the anterior choroid artery [79].

### Other surgical approaches

A combination of CSF ‘washout’ and fibrinolytic agents have also been trialled to prevent PHH. These treatments did not directly target the ChP, although they may have an indirect effect as discussed later this in review. The most promising of these results are reported in the 10-year follow-up of patients who underwent DRIFT (drainage, irrigation and fibrinolytic therapy). This did not result in a significant reduction in shunt requirement but did show a lower rate of severe cognitive disability and death [17]. In a similar vein, the ELVIS and EARLYDRAIN trials, which respectively assessed early CSF diversion in PHH in paediatrics and in subarachnoid haemorrhage, may have similar indirect effects on the ChP [80, 81].

### Long-term outcomes

The success of the DRIFT study at the 10-year follow-up point highlights our limited understanding of the long-term effects of these interventions that directly or indirectly target the ChP. This question has been raised before [82] and at present, there are no conclusive answers. At 2-year follow-up, there was no significant difference in brain growth or cognitive scores in a randomised trial of ETV + CPC versus VPS, with notable normalised brain volume loss between year 1 and 2 [83]. The aforementioned study by Pople et al., with a mean follow-up of 10 years, highlights key developmental benefits of successful CPC (where VPS is not required), such as better educational outcomes of children that did not require subsequent VPS.

Given the knowledge that choroid plexus function could play a key role in neurodegenerative and neuroinflammatory conditions [84], it is pertinent that we continue following these patients up, so that we can characterise the impact of CPC at both extremes of life.

### Future research

There are several ongoing research initiatives that continue to drive innovation in this area. DOLPHIN-UK has published a standardised protocol for neuro-endoscopic lavage through a Delphi Consensus approach, with the aim to guide and standardise future research in this field [85]. This is now being investigated in a prospective randomised clinical trial in the UK, ENLIVEN-UK (ISCRCTN14018410) [86]. The TROPHY registry was set up in 2019, collecting prospective international multi-centre data on the management of neonatal intraventricular haemorrhage (IVH) [87]. The 2-year status report demonstrates an increase in the use of neuro-endoscopic lavage by centres and the reported outcomes at 6 months show that patients who received neuro-endoscopic lavage had the lowest rate of shunt-dependence (48%), though the retrospective and non-randomised nature of these results should be considered [88]. The ESTHI trial (NCT04177914), a multi-centre RCT in North American paediatric neurosurgical centres is comparing ETV alone with ETV and CPC combined, with a predicted completion date in 2027.

## Molecular targets to alter choroid plexus function

**Fig. 2:**
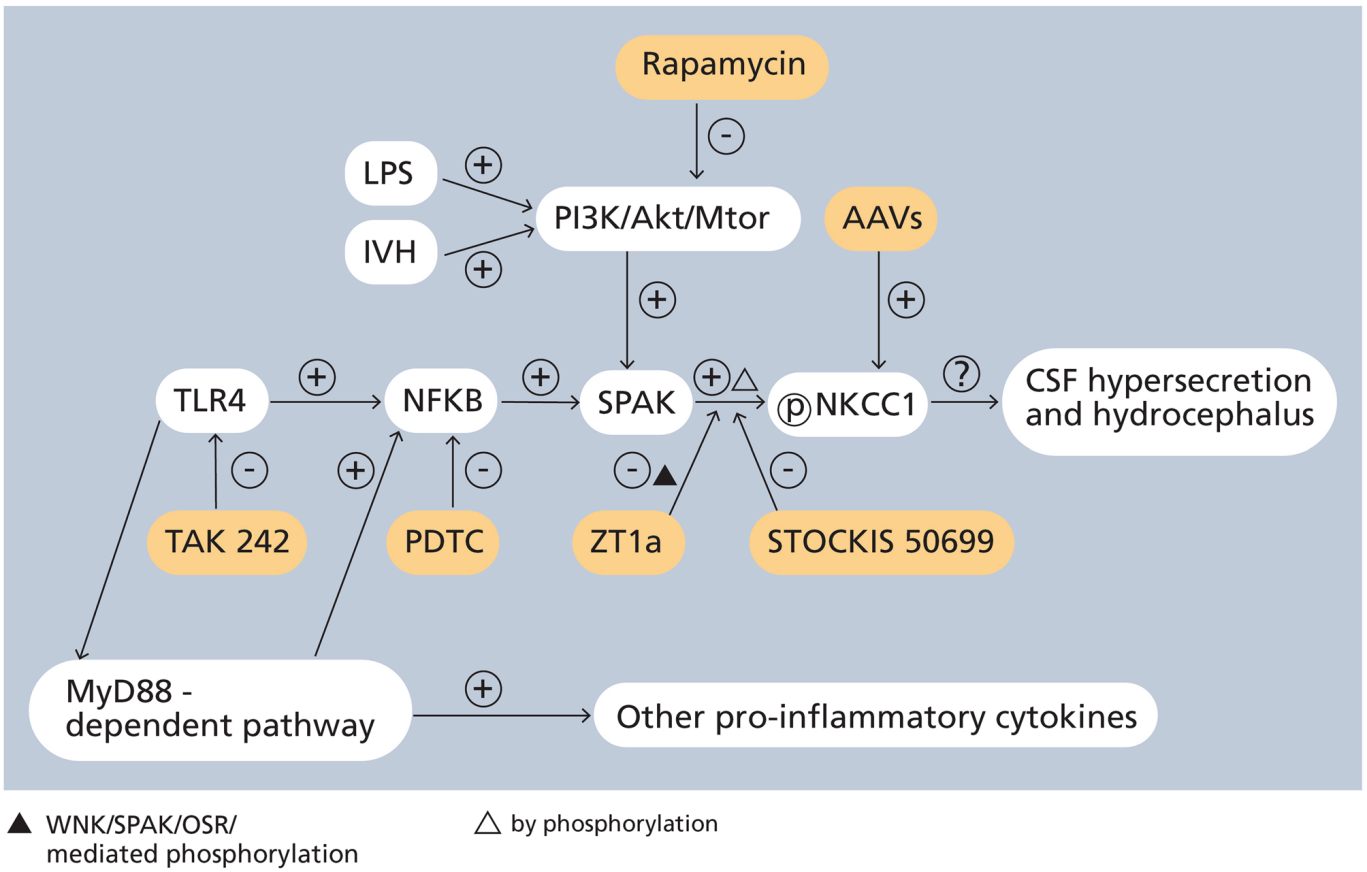
Overview of the TLR4-NKCC1 inflammatory pathway. TLR4 stimulation by inflammatory/infectious/haemorrhage related ligands leads to NFκB activation, via MyD88 dependent and non-dependent pathways. This can be inhibited with TAK 242 (TLR4) and PDTC (NFκB). This then stimulates SPAK-mediated phosphorylation of the NKCC1 channel, which increases its activity. The PI3k/Akt/Mtor pathway also feeds into this, driven by LPS and IVH. Rapamycin is an inhibitor of this pathway, whilst ZT1a and STOCKIS 50,699 can inhibit the SPAK-mediated phosphorylation. Adeno-associated viruses can also affect luminal membrane NKCC1 expression. Increased NKCC1 activity/expression/phosphorylation likely drives CSF hypersecretion and hydrocephalus, but the evidence is not fully conclusive

**Fig. 3:**
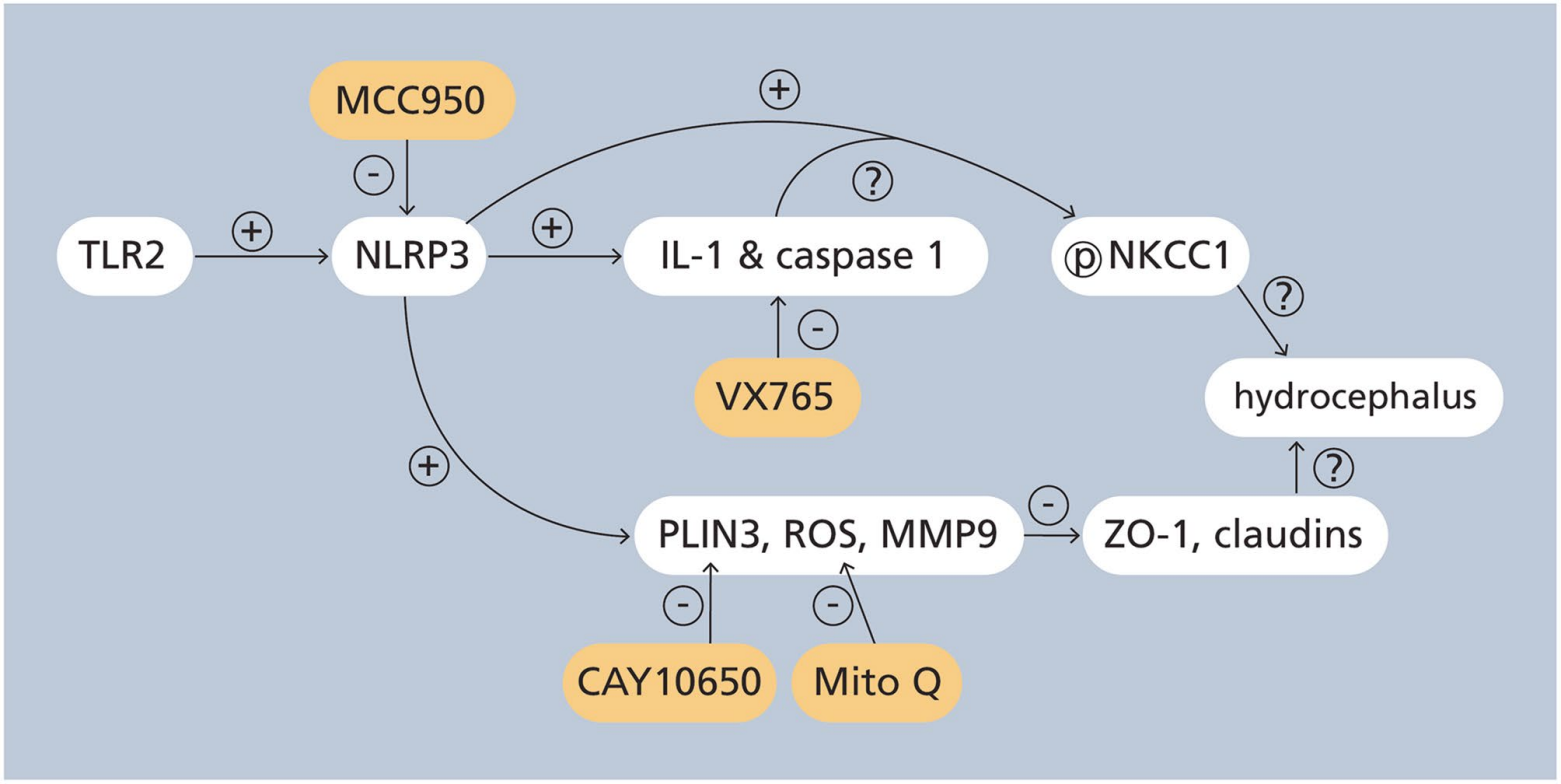
Overview of the NLRP3 inflammasome pathway. TLR2 is (one of) the upstream mediators and ligand-binding results in activation of the NLRP3 inflammasome, which can be inhibited by MCC950. NLRP3 drives IL-1 and caspase-1 activity (inhibited by VX765), both inflammatory mediators. There may be a link to NKCC1 activity, which drives CSF hypersecretion. It also leads to junctional protein disruption (ZO-1, claudins) via PLIN3 (inhibited with CAY10650), increased reactive oxygen species (ROS, inhibited with MitoQ) and matrix metallopeptidase 9 (MMP9), which will disrupt choroid plexus function. The exact interaction of these molecules is unknown, but the reactive oxygen species may drive NLRP3 activity via a positive feedback loop

**Fig. 4:**
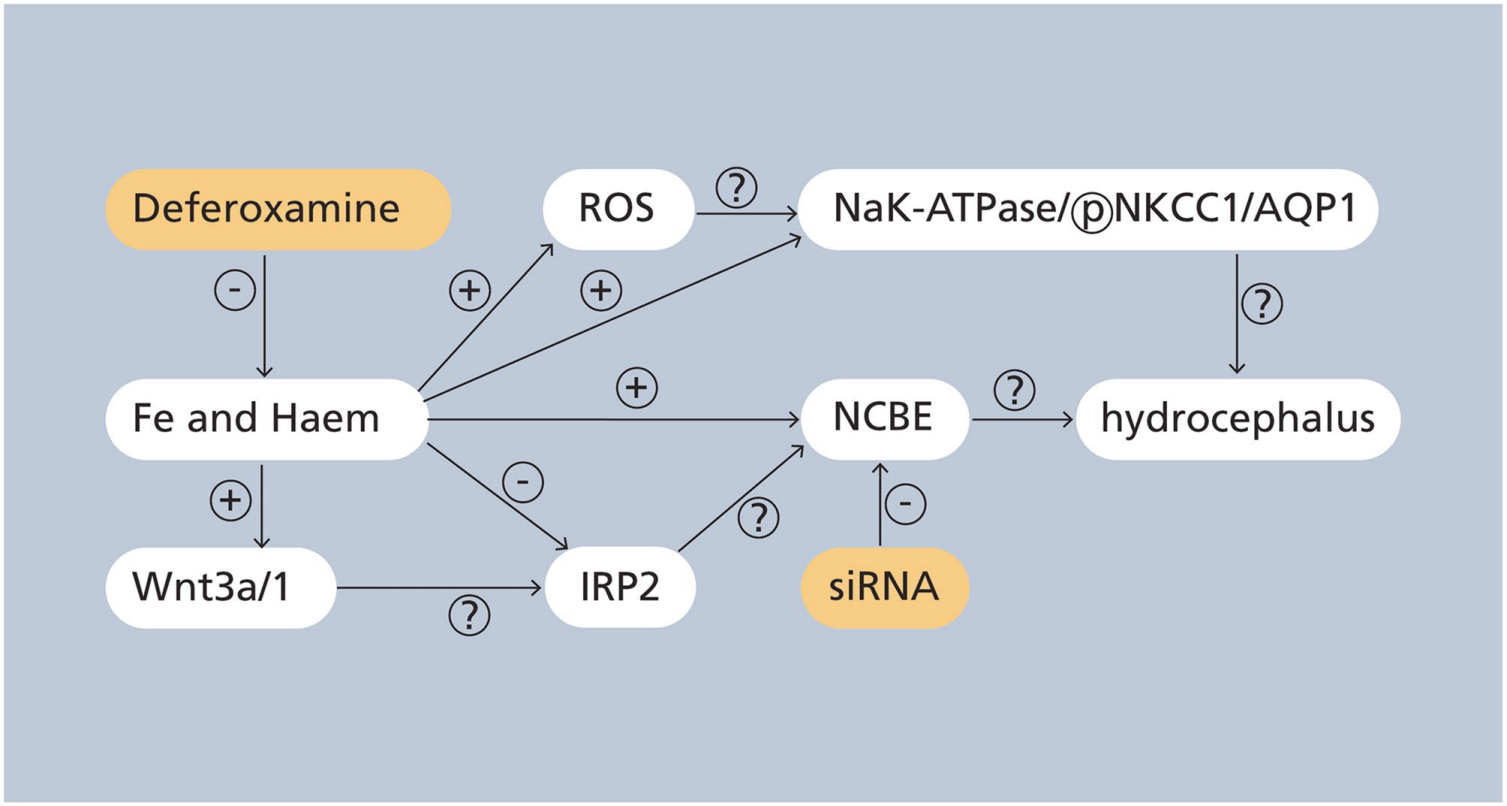
Overview of the iron/haem breakdown pathway. Deferoxamine chelates iron (Fe) and reduces the inflammatory burden of Fe and haem. These likely drive a CSF hypersecretory response through stimulation of NaK ATPase, NKCC1 and AQP1 activity in the choroid plexus, with reactive oxygen species as a possible intermediary. The pathways remain unknown, but increased levels of WNT1/3a markers were found. Iron regulatory protein 2 (IRP2) is involved, as its reduced levels are associated with increased Fe and sodium-bicarbonate cotransporter electroneutral (NCBE) activity, which is involved in CSF production. This is inhibited by iron chelation and siRNA interference. Ferroptosis, with raised levels of ROS and lipid peroxidation, has also been identified as a key marker in the ChP in the context of PHH, though whether this is cause or effect is unclear [122]

**Fig. 5:**
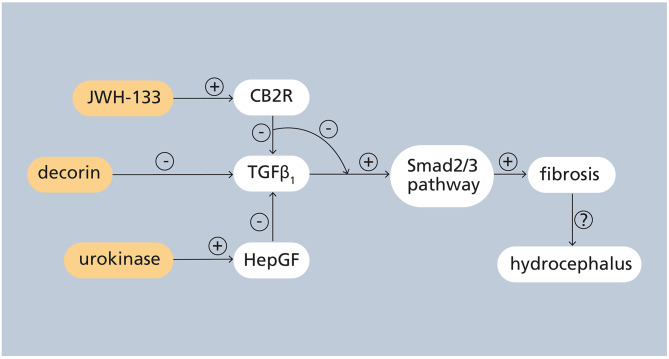
Overview of the TGFB1 and fibrosis pathway. Transforming growth factor beta-1 (TGFβ1) is considered key in driving post-haemorrhagic subarachnoid fibrosis, a key histological finding in patients with PHH. The Smad2/3 pathway is a downstream effector of this and decorin is a potent inhibitor of TGFβ1. Urokinase has also been shown to downregulate its activity, likely via hepatocyte growth factor (HepGF). JWH-133 activates the cannabinoid-2 receptor which has as similar inhibitory effect, including on the downstream pathway

**Fig. 6:**
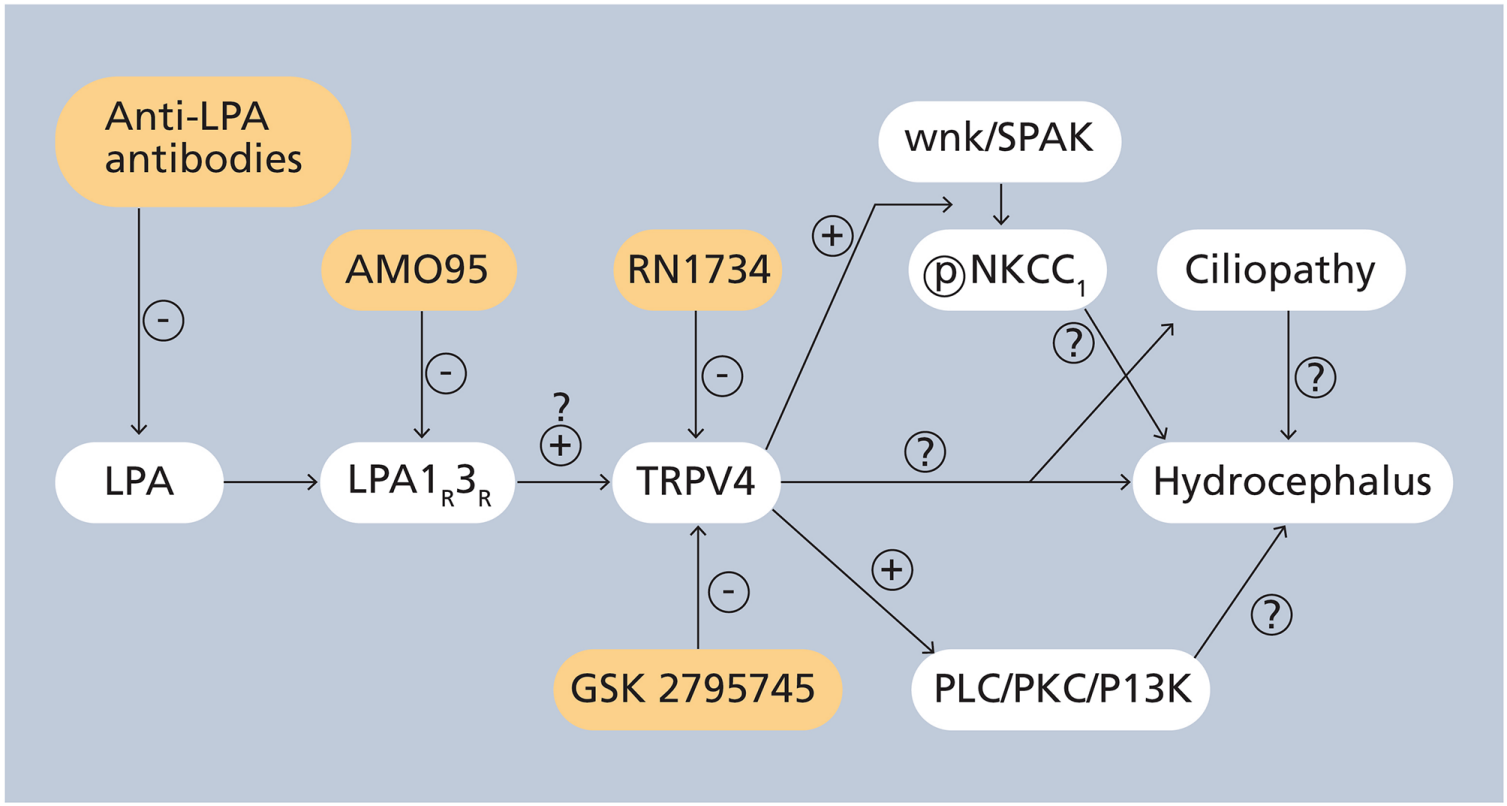
Overview of the LPA/TRPV4 pathway. TRPV4 is central in this pathway and LPA is a key activating ligand, acting via LPA1 and 3 receptors. These steps can be inhibited by anti-LPA antibodies and AMO95 respectively. TRPV4 drives CSF hypersecretion via NKCC1 activation, with which it colocalises. Wnk/SPAK mediated phosphorylation may play a role in this. PLC, PKC and PI3K are also involved in TRPV4 driven increases in trans-epithelial ion fluxes in a human ChP cell line [145]. TRPV4 activation with an agonist increases ion-flux across a porcine ChP cell line, likely by activation of calcium dependent channels [143]. In a rat model of congenital hydrocephalus (one that is orthologous to Meckel-Gruber syndrome type 3), the administration of RN1734, a TRPV4 antagonist, completely prevented the ventricular dilation that otherwise develops [144]. There may be several downstream effects of TRPV4 activation, as there are also reported links to a delayed ciliopathy that can contribute to hydrocephalus

Several molecular targets in the ChP, that are either directly or indirectly involved in its CSF production and can be used to modulate its activity, have been discovered in recent rodent models. Since the Karimy et al. publication in 2017 [89], which is described in further detail below, this field has experienced a resurgence, investigating inflammatory cascades, those involving iron processing, and more. Key agents and pathways have been summarised in Figs. 2, 3, 4, 5 and 6.

### The inflammation pathway: TLR4-SPAK-pNKCC1

NKCC1, a Na+/K+/2Cl- cotransporter, present on the luminal side of choroid plexus epithelial cells, plays an important role in CSF production. Its exact nature, in both normal and pathological conditions, remains unclear. The use of intraventricular furosemide and bumetanide lowered CSF production in the ChP [90, 91], and this has been repeated in more recent experiments [89, 92–95], including a comprehensive study on ex-vivo ChP [96]. Whilst this study suggests an outward transport direction for NKCC1, there is ongoing debate as to whether its transport direction is predominantly inward or outward [5, 97]. In addition to this, there is compelling evidence against an osmotic gradient being the driving force in CSF production (see [5, 96] for a review of the evidence), and one of the hypotheses to explain this is that NKCC1 directly transports H_2_0 molecules [98–101]. Recent work has however shown that the amount of H_2_0 that could move through NKCC1 is not sufficient to explain the volume of CSF produced in vivo [102].

Despite the uncertainty of transport direction, the nature and the extent of its contribution to CSF production, NKCC1 is a key player in ongoing research into inflammatory pathways driving CSF hypersecretion. This was highlighted by Karimy [89] in their mouse model of PHH. Through stepwise inhibition and direct measurements of CSF production, they showed a clear upregulation of inflammatory pathways, involving TLR4, NFκB and SPAK. SPAK is key in phosphorylation of NKCC1, which drives its activity [102–104]. Inhibitors targeting TLR4 (TAK-242), SPAK-NKCC1 interaction (STOCK1S-50699) and NFκB (PDTC), attenuated the hydrocephalus and CSF hypersecretion that was present in the untreated mice with IVH. Not only has this model suggested several promising pharmacological targets, but it also challenges the longstanding belief that PHH is predominantly caused by CSF malabsorption.

Later work led to the development of a specific and potent SPAK inhibitor, ZT-1a, which inhibits WNK/SPAK/OSR1 mediated phosphorylation of NKCC1, as well as other cation/Cl- cotransporters, and was effective in reducing post-haemorrhagic CSF hypersecretion [92] when administered intraventricularly in rats. Notably, it is effective in ischaemic stroke models when given systemically, presumably due to localised breakdown of the BBB, where it reduces oedema, infarct size and improves neurological outcomes [92]. This suggests that there must be a wider role for dysregulated cation transport in the pathophysiology of ischaemic stroke, which may not yet be fully understood.

The evidence supporting NKCC1’s central role in CSF secretion and its potential as a target is further strengthened by more recent mouse models with genetic knockout of either AQP1 or NKCC1, as well as viral choroid plexus-specific knockdown of either channel. AQP1 function did not significantly contribute to CSF production, but NKCC1 function did, with a 40% reduction when knocked down [105]. This study confirmed that the NKCC-1 (and AQP-1)-coding transcripts were significantly reduced up to 3 weeks but did not assess this at longer intervals.

There is growing evidence that PHH and post-infectious hydrocephalus (PIH) are both forms of acquired inflammatory hydrocephalus [23]. Robert et al. have attempted to find a common pathway to modulate these inflammatory responses [106]. Their mouse models with either intraventricular lipopolysaccharide (LPS) infusions or IVH allowed them to carry out multi-omics analysis on harvested ChP tissue. This demonstrated significant overlap and marked the PI3K/Akt/mTOR pathway, which drives SPAK activation, as a feasible therapeutic target. Rapamycin, an mTOR inhibitor, delivered systemically, attenuated CSF hypersecretion in both models. Not only did this work strengthen the notion that ChP inflammation is key in both forms of hydrocephalus, but more importantly they have found a viable pharmacological agent that is effective in both. Rapamycin and its derivatives (e.g. everolimus) have been used effectively in the treatment of advanced cancers, renal transplant rejection and subependymal giant cell astrocytoma in patients with tuberous sclerosis. The known safety-profile, side-effects and dosing regimen make it a more viable candidate for further research, but caution is advised in presuming the mechanism of action in hydrocephalus, as it has such wide-ranging effects [107].

The inflammatory cascade that lies downstream of TLR4, specifically the MyD88-dependent pathway that drives the production of pro-inflammatory cytokines [108], provides many more possible targets for pharmacological inhibition, and is not necessarily limited to CSF hypersecretion in hydrocephalus [109].

Not all evidence points towards inhibition of NKCC1 however, lest we assume this is the obvious solution. In a mouse model of both kaolin-induced obstructive hydrocephalus [110] as well as PHH [111], adeno-associated virus (AAV) induced overexpression of NKCC1 on the luminal surface of ChPECs was effective in mitigating ventriculomegaly and increased CSF clearance [111]. The authors theorise that the ChP relies on phosphorylating NKCC1 to enhance CSF clearance, possibly triggered by raised extracellular K+. The actions of NKCC1 in this context are therefore strengthened through AAV-overexpression. They also highlight that the natural decline in CSF [K+] in early postnatal development coincides with the GABA-switch, where GABA’s role changes from excitatory in early cortical progenitor cells to its classical inhibitory role [112], which is closely related to ion concentrations in the interstitium [113]. Any intervention that interferes with this process has the potential to exacerbate or alleviate several neurodevelopmental disorders [114]. It should be noted that the timeframes of their experiments differ significantly from the other reported studies and that this may contribute to the discordance in these results. For further discussion, see ‘The temporal profile of changes in the choroid plexus’.

### The NLRP3 inflammasome

There is another player in ChP inflammation, the NLRP3 inflammasome. This has been shown to play an important role in driving neuroinflammation in intracerebral haemorrhage and BBB breakdown [115, 116], and it has been investigated in a rat PHH model to assess if it has a similar effect on the ChP.

In their PHH model, Zhang et al. confirmed CSF hypersecretion, raised levels of phosphorylated NKCC1 channels, and found increased expression of NLRP3 components in ChP samples with evidence of TLR2 activation (a likely upstream mediator) and its downstream inflammatory mediators. By blocking NLRP3 with MCC950, which downregulates caspase 1 and IL-1β, they were able to attenuate the CSF hypersecretory response and reduce the levels of inflammatory mediators [93]. Interestingly, they also assessed ChPE after intraventricular bumetanide, which is commonly used to determine whether hypersecretion is NKCC1 related and found that this reduces NKCC1 phosphorylation as well as CSF secretion [93]. This highlights that there may be secondary effects in these and previous experimental interventions that need to be considered in the interpretation of results.

The downstream effects of NLRP3 upregulation led to an increase in lipid droplet formation in ChPECs’ mitochondria in their PHH model, with associated junctional protein disruption, such as ZO-1 and claudins [94]. A downstream effector of this was possibly PLIN3, in conjunction with increased reactive oxygen species (ROS) and MMP9 activity. They investigated PLIN3 involvement with NLRP3 -/- models and direct PLIN3 inhibition with systemically administered CAY10650. Both knockout models and PLIN3 inhibition successfully attenuated CSF production in their models. These authors infer a positive feedback loop of ROS driving further NLRP3 activation, which can be disrupted by MitoQ.

Related to this work, caspase-1 was targeted in a subarachnoid haemorrhage model of rats, where inhibition with intranasal VX765 led to improved neurological outcomes and reduced hydrocephalus [117].

Whilst these pathways are highly complex and only partially discovered there is clear scope for continued intervention development. The overlap with the TLR4-SPAK-NKCC1 pathways investigated by Karimy and others remains unclear, but the results mentioned above suggest several systemically administered promising therapeutic agents.

### The use of intraventricular stem cells

Another treatment option exploits the ability of stem cells to modulate inflammatory responses. Ahn et al. in 2014 investigated the impact of intraventricularly injected mesenchymal stem cells (MSCs) in a rat IVH model [118]. They found clear improved neurological outcomes and attenuated hydrocephalus. Whilst not specifically assessing the ChP, they did report that the MSCs appeared to lower the levels of multiple inflammatory cytokines in the CSF and the degree of ventricular dilatation at 28 days post-injury. They postulate that the wider, multi-modal anti-inflammatory properties of MSCs [119, 120] may make them a more capable therapeutic agent. Intraventricular administration of MSCs in a rabbit pup model of IVH [121] also alleviates hydrocephalus. This work analysed the impact on specific brain structures, including the ChP. The use of MSCs restored transforming growth factor beta (TGFβ), CTFG and MMP9 levels as well as the expression of ChP AQP1 and AQP4 in ventricular wall ependyma. The authors also investigated whether there was a demonstrable impact on TLR/NFκB signalling pathways but found no evidence thereof.

Recruitment of endogenous neural stem cells with granulocyte-colony stimulating factor (G-CSF) has also been investigated in a rodent IVH model [122]. Systemically administered G-CSF or neuroprotective lithium chloride (LiCl) [123], reduced the incidence of PHH, with combination therapy having the best outcome. There was evidence of reduced apoptosis, though no specific analysis of the downstream effects of either G-CSF or LiCl were carried out.

### Iron and other blood-breakdown products

The earliest point of intervention in the disease process of PHH, bar preventing the initial bleed, is to address the harmful effects of the blood breakdown products, such as iron [124], with the aim to reduce its impact on the ChP and other CNS structures. Scope for surgical intervention in this space was discussed previously, but molecular targeting of this pathway is also under investigation.

Several research groups have looked at the use of iron chelation with deferoxamine. Rat models involving IVH, haemoglobin, or separate intraventricular iron injections [125–127] lead to hydrocephalus. The use of protoporphyrin IX, an iron-deficient haem-precursor, did not have this effect, suggesting iron is a causative agent [126]. These experiments demonstrated increased levels of WNT1/WNT3a pathway markers [125], which is linked to fibrosis in other organs [128]. The concurrent administration of deferoxamine drastically reduces the rates of hydrocephalus and lowers the iron concentrations in CSF and the WNT1/3a activity levels [125]. Importantly, delayed administration of deferoxamine is also effective [129]. Looking more closely at the ChP, both models of germinal matrix haemorrhage (GMH) or FeCl_3_ injection demonstrated reduced levels of iron regulatory protein 2 (IRP2) and increased NCBE in the ChP in conjunction with CSF hypersecretion [130]. Both the use of iron chelation and siRNA to interfere with NCBE/NBCn2 (Slc4a10) expression attenuate these changes suggesting that excess iron may be a driving factor, with IRP2 as an intermediary. Other work has demonstrated increased expression of Na/K-ATPase, NKCC1 and AQP1 [127] under similar conditions. This also highlighted the potentially transient nature of these effects – whilst a significant proportion of treated rats did not develop hydrocephalus for 11 days, this did develop at 21 and 60 days.

Ferroptosis, with raised levels of ROS and lipid peroxidation, has also been identified as a key marker in the ChP in the context of PHH, though whether this is cause or effect is unclear [131]. Iron metabolism within the CNS environment is a complex process in normal and abnormal physiological conditions, with significant scope for future research [132].

Other components of blood can also trigger pro-inflammatory responses after IVH, such as peroxideroxin-2, the most abundant protein in erythrocytes after haemoglobin. When present intraventricularly, it is pro-inflammatory and induces hydrocephalus in a rat model [133]. It leads to active recruitment and migration of macrophages in the ChPE. By targeting recruited macrophages, through the injection of clodronate liposomes (which are taken up by the macrophages and lead to their death), the hydrocephalic response can be attenuated.

### Transforming growth factor Beta-1 and fibrosis

Transforming growth factor Beta-1 (TGFβ1) has been of interest since it was found to be elevated in both animal and human studies of CNS injury [134–136], for example after SAH. It follows a biphasic pattern in SAH, especially in patients that develop hydrocephalus where an initial peak, attributed to release from platelets, is followed by a delayed, higher and more sustained peak, attributed to immune cell infiltration and an activated ChP epithelium [135].

TGFβ1 and its associated Smad2/3 pathway are involved in fibrotic processes throughout the body and given the findings of subarachnoid fibrosis in PHH [137] and PIH, it was explored as a therapeutic target by several research groups. Initial studies trialling TGFβ blockade with pirfenidone, losartan, colchicine and decorin found no success in preventing hydrocephalus in rodent models [138, 139]. Decorin is a potent inhibitor of TGFβ1, acts on extracellular matrix (ECM) components and other growth factors and has anti-tumorigenic properties. Its effects were investigated again in later work and a prolonged infusion (over 14 days) in a rat model was successful, in abolishing a kaolin-induced hydrocephalus [140]. Decorin had spread throughout the CNS leading to reduced Smad2/3 phosphorylation and reduced fibrosis/inflammation in the subarachnoid space. Subsequent diffusion tensor imaging demonstrated that the use of decorin mitigates hydrocephalus related CNS injury [141]. Similarly positive results were achieved with two intrathecal injections of decorin, with evidence of the TGFβ/Smad pathway being inhibited [142]. These results are at odds with prior work [139], where two injections of decorin did not improve the outcomes. This may be related to the timing of decorin injection and the age of the rats. Yan et al. administered decorin immediately prior to intracerebroventricular (ICV) injection of blood and at day 10 whereas Hoque et al. administered it immediately after the ICV injection of blood and at day 5.

Other approaches to inhibiting TGFβ1-driven fibrosis include the use of intraventricular urokinase [143], which has been shown to promote activity of hepatocyte growth factor, which regulates TGFβ1 expression [144]. In a model of kaolin-induced hydrocephalus, this attenuated ventricular dilation and inhibited deposition of ECM molecules. The use of a kaolin-induced hydrocephalus model removes any contribution of a fibrinolytic effect on intraventricular blood products and highlights that urokinase can have wider-ranging positive effects in this context. Human trials have not reflected these results when urokinase is used for IVH, which suggests that TGFβ-driven fibrosis may play a less crucial role than in these kaolin-induced hydrocephalus models.

Tan et al. also investigated the cannabinoid receptor 2 (CB2) as a therapeutic target in an IVH model. Agonists at CB2 attenuated PHH through a reduction in fibrosis and TGFβ1 downregulation, which could be inhibited by a CB2 antagonist [145].

The fibrosis pathway is clearly an additional promising therapeutic target and shows that we should attribute a significant role to subarachnoid fibrosis causing impaired CSF circulation and reabsorption in PHH and PIH. It is still unclear whether this pathway also has a direct effect on the ChP and if its involvement needs to be considered in future therapies.

### TRPV4 and lysophosphatidic acid

Lysophosphatidic acid (LPA), which is known to worsen neurological outcomes in haemorrhagic events [146] can induce hydrocephalus [147]. LPA may be one of the blood components that drives PHH, as it is present in platelets and bound to albumin [148] and its intraventricular administration causes chronic hydrocephalus [149]. LPA receptors 1 and 3 are key in this pathway, demonstrated with knockout mice and the LPA1R inhibitor AM095. The authors noted evidence of ependymal wall disruption and attributed the hydrocephalus to cilia dysmotility [149]. Anti-LPA antibodies have been used in traumatic spinal cord injury mouse models and may be a potential therapeutic agent [146].

The TRPV4 channel is widely expressed in many organ tissues [150], including the ChP. It is purported to mainly be involved in calcium influx. It can be activated by osmotic changes, pressure changes and direct mechanical stress [151]. Its activation with a TRPV4 agonist increases ion-flux across a porcine ChP cell line likely by activation of calcium dependent channels [152]. In a rat model of congenital hydrocephalus (one that is orthologous to Meckel-Gruber syndrome type 3), the administration of RN1734, a TRPV4 antagonist, completely prevented the ventricular dilation that otherwise develops [153]. PLC, PKC and PI3K are involved in TRPV4 driven increases in trans-epithelial ion fluxes in a human ChP cell line [154].

There appears to be a link between LPA and the TRPV4 channel in the development of PHH. Raised levels of LPA levels were found in human CSF after SAH as well as in rat models of PHH. Intraventricular LPA replicates the development of PHH in rats and was used to investigate its downstream effectors [147]. These experiments demonstrated that LPA can act as an agonist of TRPV4 and lead to raised intracranial pressure (ICP) and CSF production. With the intraventricular administration of the TRPV4 inhibitor RN1734 these effects were alleviated, and they were aggravated by an agonist (GSK1016790A). Inhibition of TRPV4 alleviates hydrocephalus and CSF hypersecretion, whereas activation worsens this. TRPV4 also colocalises with NKCC1 and TRPV4 modulation alters NKCC1 mediated ion flux, likely through a calcium-mediated WNK/SPAK pathway. Blocking TRPV4 activation (RN1734) or NKCC1 with (bumetanide) in healthy rats had no impact on CSF production in a separate study, suggesting their effects may only be significant in a pathological state [155]. However, as both drugs were given intraperitoneally, bumetanide does not cross the blood-brain-barrier (BBB) [156], and the BBB penetrance of RN1734 is not known, alternative explanations for this outcome are possible. Indeed, when delivered directly into the ventricle, bumetanide and the more potent derivative furosemide certainly do inhibit CSF production in normal mice [96].

A promising agent in this field is GSK2798745, an orally active TRPV4 channel blocker, which is shown not to cause significant side-effects in humans in a placebo-controlled study assessing its pharmacokinetic profile [157], making it a suitable agent for future trials.

Crucially, LPA is also believed to serve as a channel modulator for TRPV4 with a functional outcome similar to an agonist at TRPV4 [147] As these authors mention in their discussion, this may represent a parallel pathway in the development of PHH, working alongside and perhaps amplifying the inflammatory cascades described by Karimy and others [23, 106, 158] given they both lead to increased NKCC1 activity. The TRPV4 pathway may represent a more immediate effect, followed by the inflammatory cascade. Toft-Bertelsen et al. hypothesise that there is also a delayed ciliopathy through TRPV4 agonism, which could explain the findings of Lummis et al. [149] and supplement pathological findings in hydrocephalus beyond the ChP. Here, ependymal changes were noted from 3-hours post-insult and rapidly evolved over 24 h, though we should be reluctant to directly compare timescales across experiments.

### Aquaporins

As a ubiquitous water channel, aquaporins have been a key target in the investigation of CSF production. Only a limited number of studies have managed to target these channels in the direct treatment of hydrocephalus, but there is wider supporting experimental evidence.

The role of AQP1 in CSF production by the ChP continues to be debated, despite its widespread expression in the choroid plexus [5, 33, 39]. Some data suggests a significant reduction in transcellular water permeability across the ChPE in cells lacking AQP1 in a knockout model [159, 160], whereas more recent work by Jensen et al. question its contribution [105], demonstrating that in both knock-out models and viral choroid-plexus specific knockdown of AQP1 there is no change to CSF production. Methods and mice used across both studies are similar and both report changes in permeability in AQP1 knock-out. The reported CSF-production rates differ significantly between both studies (Oshio et al.: 0.30 and 0.38 µl/min for knock-out and WT mice, Jensen et al.: 0.72 and 0.78 µl/min respectively). There is further discrepancy in reported ICP measurements, as well as analytical methods of permeability and monitoring during anaesthesia [105], which may explain the contradictory results.

In AQP1 knockout mice, ventricular volumes are slightly smaller, both at baseline and in a kaolin-induced model of hydrocephalus [161]. It appears that in that model, WT mice’s AQP1 levels may reduce by means of endocytosis, as a possible compensatory mechanism. Forskolin stimulation of CSF production is also reduced by 25% in AQP1 knockout mice [159].

In certain pathological states, AQP1 and AQP4 expression is increased, such as in rat models of subarachnoid haemorrhage with hydrocephalus [162] and in choroid plexus papilloma, where resultant communicating hydrocephalus is related to higher AQP1 expression and lower expression is not [163]. Differential localisation of the AQP1 channels has been demonstrated to differ in rodent models of both obstructive and communicating hydrocephalus, where a shift towards the stromal and basolateral side of the choroid plexus epithelium was noted, compared to a predominantly apical distribution in healthy rats [164]. A similar shift has been demonstrated in the choroid plexus of a pre-term infant with obstructive hydrocephalus due to a type 2 Chiari malformation [165]. This may represent an adjustment to altered CSF dynamics that could reduce the rate of CSF production in the choroid plexus, or even invert this and drive absorption.

The results of knock-out models are difficult to interpret, as AQP1 is a ubiquitously expressed channel, with effects on renal function and central venous pressure, which can result in a reduction in ICP [159, 166]. There may be additional compensatory mechanisms during the embryonic development of these mice that could further complicate the interpretation of these results. Humans with non-functional AQP1 mutations have a reduced renal urine concentrating ability [167], but do not present with clear neurocognitive phenotypes or symptoms suggestive of intracranial hypotension. With some evidence that apical relocation of AQP1 channels occur in models and human disease, altered AQP1 levels may be a consequence rather than a cause of hydrocephalus.

To experimentally determine whether AQP1 is a meaningful target in the treatment of hydrocephalus, is challenging due to a limited understanding of AQP1 gene regulation and the absence of useable non-toxic specific inhibitors. Mercury chloride and tetraethyl-ammonium are non-specific inhibitors of AQP1 but are not viable pharmaceutical agents due to toxicity [168]. The inhibitory effects of acetazolamide and furosemide on AQP1 [168] are difficult to separate from parallel inhibition of carbonic anhydrase and NKCC1. Downregulation of AQP1 has been achieved in cultured rat ChPECs, using curcumin, but no data was reported on CSF production, and it is difficult to separate the effect from parallel inhibition of Na+/K+ ATPase [169]. When AQP1 levels were reduced to 30% of normal in rat brain using an siRNA-based experimental approach targeting a key transcription factor, CSF production was reduced by 19% [170] but more recently, in the aforementioned study by Jensen et al., an adeno-associated virus ChP specific knock-down of AQP1 did not significantly alter CSF production by the choroid plexus [105].

It remains unclear whether AQP1 is a key player, contributor or a meaningful target in the treatment of hydrocephalus, given the range of seemingly contradictory experimental results. This uncertainty stems from differences in methodology, the lack of disease-specific models and the inability to directly assess AQP1 function with an inhibitory agent. To meaningfully determine the feasibility of AQP1 as a therapeutic target, experimental designs must account for the developmental compensation that may occur in knock-out mice and closely mimic the pathological processes we are seeking to treat, such as those of intraventricular haemorrhage.

### The temporal profile of changes in the choroid plexus

The temporal profile of the molecular changes and targeted interventions that have been discussed is not yet clearly defined. A detailed understanding of this is required to guide the timing and duration of any intervention designed to alter the course of this pathology.

Most referenced experiments rely on invasive procedures to measure the impact of interventions on CSF production and the levels of inflammatory cytokines in the immediate period after the modelled cerebral insult, which results in limited ‘long-term’ data, especially when animal sacrifice is required for data acquisition, such as choroid plexus immunohistochemistry. There is significant variability in the timing of this assessment, either taking place within 24 h to 7 days after the modelled insult [89, 93, 94, 106, 133, 147], or 1–4 weeks post-intervention [118, 122, 125, 129, 130, 138–140, 144]. Notable exceptions [111, 127] include Sadegh et al., who evaluate their mice at 2–3 weeks and at 6–9 weeks post-insult and intervention, with non-invasive and invasive methods. Their results focus on the noted changes in CSF clearance and compliance, suggesting that a transient hypersecretory response may lead to such longer-term changes in CSF dynamics. It is perhaps no surprise that their seemingly contradictory approach (NKCC1-overexpression instead of inhibition) leads to such different results, given the discrepancy in timeframes amongst these studies.

There is also variability in the timing of intervention, with either concurrent administration of treatment at the time of insult, or administration just prior or afterwards. To the authors’ knowledge, no studies have assessed the impact of significantly delayed administration of such interventions, with the longest interval reported as 72 h [129].

For a patient who has sustained an intraventricular haemorrhage and subsequently develops hydrocephalus, it is not the immediate imbalance in CSF production and absorption which requires *novel* treatment, but the long-term, chronic imbalances that do. We have several effective treatment options to manage an immediate excess of CSF, such as a lumbar puncture or external ventricular drain. A significant proportion of patients develop chronic hydrocephalus despite such interventions, as they do not address the root cause of that chronic process.

It is therefore pertinent that we do not measure the success of novel treatments by their ability to reduce the acute excess production of CSF, but instead by their long-term impact on imbalances in CSF dynamics, as it is those patients that will benefit most from innovation. Based on the evidence currently available, we cannot yet conclude whether these inflammatory and hypersecretory changes in the choroid plexus are a transient feature of a chronic pathological process, or whether they persist and drive this process. Tailored experiments that explore these questions are a key next step. These should also guide whether the proposed novel approaches require sustained courses of treatment or only in the acute setting.

## Barriers to translation

With such a breadth of research on therapeutic targets for hydrocephalus in rodent models, it is striking that current clinical practice does not yet reflect this. Several barriers still exist before these studies can achieve a meaningful, clinical benefit for paediatric and adult patients with hydrocephalus.

### Large animal models

Besides rodent models, of which most are in mice and rats, studies of hydrocephalus have also been performed in higher order small animal models [171–174]. To our knowledge, none of the discussed therapeutic targets have yet been explored in large animal models of hydrocephalus, which would be the next step in translating this into clinical practice.

Other fields of translational research have already been exploring molecular therapies in porcine models, such as in neurofibromatosis type 1 (NF1) [175]. Porcine models have also been used to successfully develop the use of convection enhanced delivery for the treatment of diffuse intrinsic pontine gliomas [176].

The longevity of these animals allows more longitudinal studies, their size and physiology are more comparable to humans allowing more accurate assessment of drug pharmacokinetics and dosing strategy, and surgical intervention and replication of current clinical practice is more feasible and does not require highly specialised miniaturised equipment as it would for smaller animals. The central nervous system of a pig is far more comparable than those in rodent models. They have a similar brain weight, are gyrencephalic, have a similar cortical and subcortical structure to humans, and comparable pattern of neurodevelopment that makes them ideal for experimental research [177]. Pig models allow more detailed assessment of locomotor function, behavioural changes and cognitive ability. Presumably for these reasons, key historic experiments were carried out in large animals, by Dandy and Milhorat in dogs [41] and monkeys [50] respectively. Since these landmark experiments, experimental work has shifted focus to small animal models.

The definition and correlation of ‘short-term’ and ‘long-term’ changes between a small-animal model and a human disease process is also difficult to establish and may be more closely aligned in larger animal models. Whilst there is agreement on the correlation between a rodent model’s age and specific life stages in human development, the relative timescales can change when we model the development and progression of a disease, especially in the setting of an acute insult that can result in a chronic disease.

### Canine and feline models

**Fig. 7:**
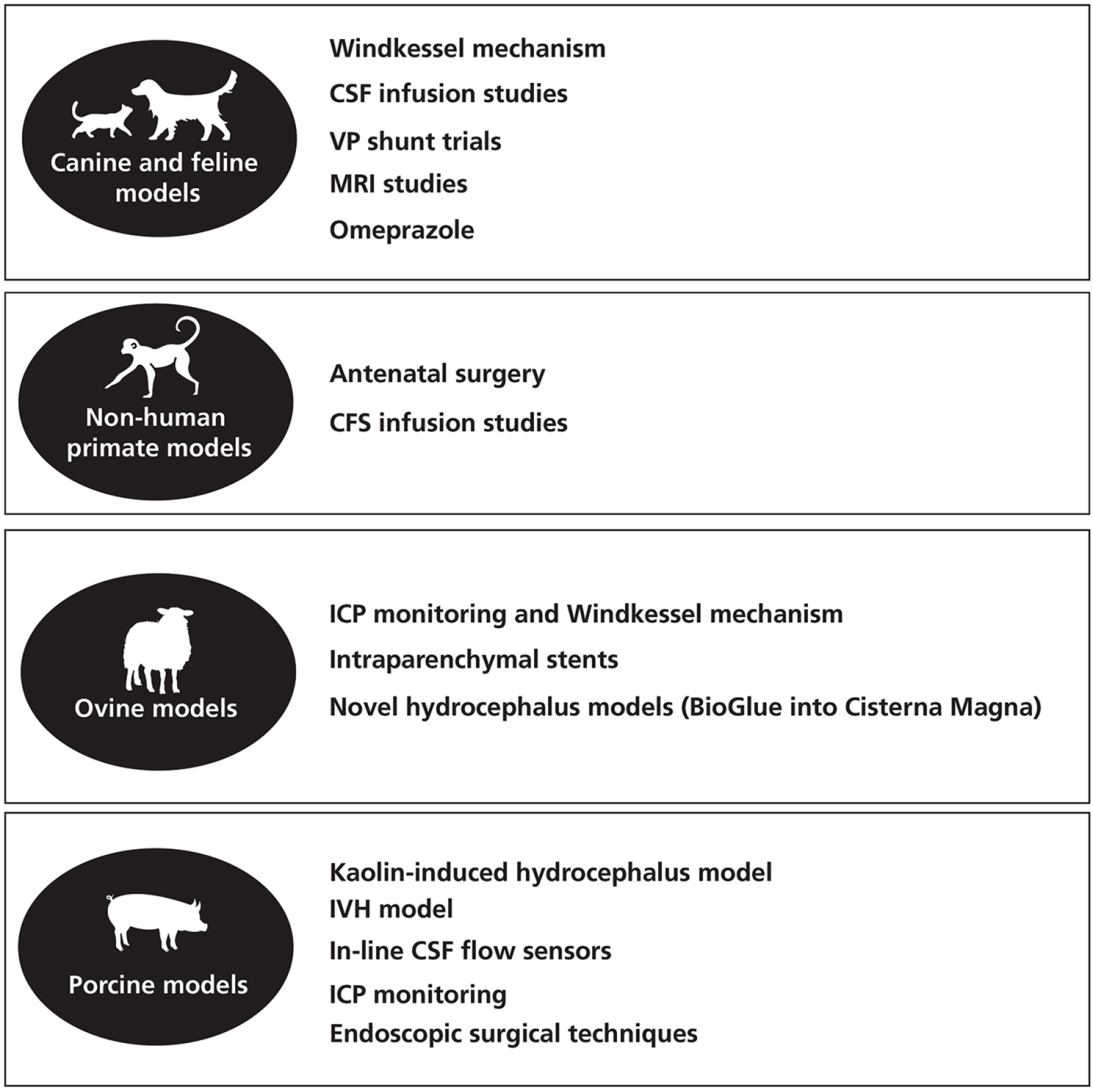
Overview of the major large animal model experiments in hydrocephalus. Relevant species together with the principal achievements in the hydrocephalus field utilising these models. Details provided within the text

There are examples of larger animal models being used over the past 20 years, with an overview shown in Fig. 7. Veterinary work in dogs and cats developing hydrocephalus treated by ventriculoperitoneal shunting suggests similar complications over similar time frames, and similar inadequacies of pharmacological treatments, providing a useful model to the human condition [178, 179].

Eskandari [180] was able to confirm the persistence of astrogliosis following ventriculoperitoneal shunt or Omaya reservoir intervention in kittens, which would be challenging in smaller animals. Klarica et al. [181] were able to infuse hyperosmolar solutions into the ventricular system of cats to demonstrate compensatory water movement from the blood which drives up ICP without maintaining a hyperosmolar state long term, by using continuous ICP monitoring. Combined data on arterial pressure pulse, ICP, CSF withdrawal and infusion across the same dogs were used to describe and refine the cerebral windkessel mechanism providing correlative data to humans not available from rodents [182, 183]. The wealth of additional data accessible from larger species also allows connections to be made between the cardiovascular system and its responses in hydrocephalus [184], and for long held expectations about pressure gradients to be revisited [185]. Cho et al. [186] show that the CSF flow rate and direction can be discerned from MRI sequences in dogs using methods developed for humans, allowing assessments of the impact of hydrocephalus and its experimental treatments on CSF velocity. Iyer [187] developed a canine model of shunt infection for use in assessment of new shunt technologies. Larger CSF volumes also allow changes in the CSF proteome in treated and untreated hydrocephalus to be assessed by pairwise comparison in dogs [188]. Intrathecal and intravenous omeprazole was shown to reduce CSF production in dogs leading to the common treatment of the condition with the drug in veterinary practice [189, 190].

New technological advancements in the field may also benefit from rigorous testing in large animal models, such as the impedance sensor providing feedback control of cerebral ventricular volume developed by Linninger using dog brain tissue [191].

### Non-human primate models

In non-human primates McCully [192] developed a method for collecting CSF samples reliably and safely with lines that remained patent over a considerable timeframe (> 1 year), while others have used the non-human primate model to investigate antenatal surgical approaches to hydrocephalus [193]. In the case of non-human primates, these models offer bipedal locomotion and an arrangement of the ventricular system most comparable to humans. Notably these species (cats, dogs, non-human primates and horses) have special protections in many countries involving legislative frameworks around research involving animals.

### Ovine models

Ovine hydrocephalus models replicate human pathological observations such as corpus callosum thinning, astrogliosis and microgliosis, and develop many of the same complications after shunting [194]. These similarities allowed testing of a wireless pressure monitoring device [195], and a low-frequency ultrasound device designed to unblock occluded shunts [196]. Santangelo [197] showed that intraparenchymal stents as an alternative to ventricular catheters could achieve resolution of kaolin-induced hydrocephalus in sheep. In addition, novel methods of establishing hydrocephalus have also been developed in this species using cisterna magna BioGlue injection, instead of kaolin, to produce moderate-severe ventriculomegaly without microgliosis [198]. Others have utilised sheep to demonstrate communication between different volume compartments involved in maintaining CSF volume [199] and that extracranial lymphatics contribute to CSF absorption, possibly as a primary pathway [200].

### Porcine models

Aquilina [201, 202] developed a neonatal pig model of IVH and showed the model replicated clinical features of the human condition including ventriculomegaly and subarachnoid fibrosis. This model also allowed the use of ultrasound imaging, shunt placement, and assessment of longitudinal changes in ICP and CSF outflow resistance. More recently, McAllister et al. published their work on a pig model of kaolin-induced hydrocephalus [203], demonstrating its strength as a model of human disease with reactive astrogliosis, inflammatory cytokines in CSF and damage to the hippocampus and subgranular zone leading to a reduction in neuronal precursor cells. Porcine models have also been used to test technological innovations such as in-line flow sensors within shunts that may offer early warning of failure [204], new designs for endoscopy equipment [205], bioimpedance detection for clinical monitoring [206], new shunt design and placement approaches [207], ICP sensors [208–211], and a cardiac-gated intracranial pulsating balloon pump that could be shown to reduce ICP [212]. Swine models are also gaining usage for studies of CSF dynamics [213].

The importance of large animal models in our understanding of CSF dynamics and production, and in the development of novel treatments for hydrocephalus cannot be overstated. As research practices move to less neurodevelopmentally sentient models, the continued relevance of these models remains clear from the available data and methodological approaches achievable in these species, with large animal progression a highly promising next step in this field of translational hydrocephalus research. As alluded to in the introduction to this section, molecular therapeutic targets have not yet been explored in large animal models of hydrocephalus, and some consideration needs to be given to why this might be.

### Further barriers to translation

The quantity of large animal models in experimental device development highlights their importance in the translational progression to human trials. Further barriers to this likely include financial, regulatory, risk-related and academic factors. These are summarised in Table 1.

The development and running costs of the facilities required to facilitate large animal models within this field are high and rely on a combination of academic and industrial funding. A wide range of specialist input is required in this type of research, and the importance of industrial funding cannot be understated. No current animal model entirely accurately reflects hydrocephalus in humans, which further complicates the translational research required. A multi-disciplinary and collaborative effort is required to drive forward and sustain academic progress in this field.

Regulations around the use of larger animal models are logically stricter. The organisation and ethical approval for this type of research presents additional hurdles and potential points of failure that researchers and institutions may be reluctant to pursue. Several of the therapeutic avenues that have been discussed involve the use of novel gene therapies. The regulatory landscape of introducing these into clinical practice is highly complex. International variations in these regulations will provide further barriers to promoting collaboration, where this is key in driving the research forward.

Another factor may be the lack of agreement within this field. As this review has highlighted, there are numerous pathways with several therapeutic agents each. Any of these agents alone may prove to be the correct treatment paradigm for our target patient population, yet we are uncertain as to which one. The lack of an obvious ‘front-runner’ may be amongst the reasons that there has not been a concerted translational effort forward, as the resources and academic opinions are divided. Perhaps a concerted, multi-faceted approach may be required to address this.

**Table 1:** Important barriers to translational research targeting the choroid plexus

• Increasingly expensive to run large animal studies	
• Strict regulations around animal studies	
• The regulatory landscape of introducing novel gene therapies into clinical practice is complex	
• Lack of academic consensus with several potential therapeutic targets	
• Pathophysiology remains poorly understood	
• Ultimately a heterogenous condition that may require a wide range of treatment paradigms	

## Conclusion

The evidence-base to use these novel approaches to target choroid plexus function is highly promising. There is now a wide array of molecular agents that may act as therapeutic targets. Consistent positive results achieved in small animal models provide ample of scope for future research.

A question that remains unanswered is whether these changes are transient and whether the effectiveness of these novel approaches is permanent. We have sought to bring together numerous studies into a comprehensive set of pathways, but it is unrealistic to draw meaningful conclusions as to the temporal profile of the changes that take place within the choroid plexus epithelium, due to the varied timescale and assumptions within the reported studies. Future work to explore this profile and that of these interventions is key to aligning these pathways.

Given the complex interplay of molecular pathways and concurrent pathophysiological processes, future research should also investigate the use of combination therapies in treating hydrocephalus. Inhibiting multiple pathways in the same model may produce a synergistic or additive effect which may be pathophysiology dependent. Similarly, an intraventricular route of administration may help reduce systemic and local side-effects of therapeutic agents and improve its end-organ efficacy.

Knowing the clinical, social and economic burden of hydrocephalus and our current approach to its treatment, we should acknowledge the importance of the advancement of this research. Despite promising results and significant conceptual advances, no targeted agents have yet been trialled in large animal models. We cannot expect translation into clinical practice before this happens, and it is encouraging that large animal models for hydrocephalus are now being developed [203]. Not only surgical but also pharmacological and molecular therapies should be actively investigated in these models in the development of a better treatment for patients with hydrocephalus.

## Data Availability

The literature search that was carried out as part of this literature review is available from the corresponding author on request.

## Abbreviations

AE2: Anion exchanger 2
Akt: Protein kinase B
AQP: Aquaporin
BBB: Blood brain barrier
BCSFB: Blood CSF barrier
ChP: Choroid plexus
ChPE: Choroid plexus epithelium
CHPECs: Choroid plexus epithelial cells
CSF: Cerebrospinal fluid
CTFG: Connecting tissue growth factor
DRIFT: Drainage, irrigation and fibrinolytic therapy
ECM: Extracellular matrix
ETV: Endoscopic third ventriculostomy
EVD: External ventricular drain
GABA: Gamma-aminobutyric acid
ICP: Intracranial pressure
ICV: Intracerebroventricular
IL-1B: Interleukin-1 beta
IVH: Intraventricular haemorrhage
KCC: Potassium chloride cotransporter
LPA: Lypophosphatidic acid
MMP9: Matrix metallopeptidase 9
mTOR: Mechanistic target of rapamycin
myD88: Myeloid differentiation primary response 88
N-KA: Sodium-potassium atpase
NBCe2: Sodium-bicarbonate cotransporter electrogenic 2
NCBE: Sodium bicarbonate cotransporter electroneutral
NF1: Neurofibromatosis type 1
NFKB: Nuclear Factor kappa-light-chain-enhancer of activated B cells
NKCC1: Sodium-potassium co-transporter 1
NLRP3: Nucleotide-binding Oligomerization Domain, Leucine Rich Repeat and Pyrin Domain Containing 3
OSR1: Odd-skipped related transcription factor 1
PHH: Post-haemorrhagic hydrocephalus
PI3K: Phosphoinositide 3-kinase
PKC: Protein kinase C
PLC: Phospholipase C
PLIN3: Perilipin 3
siRNA: Small interfering RNA
SPAK: STE20/SPS1-related Proline-Alanine-rich Kinase
TGFβ: Transforming growth factor beta
TLR2: Toll-like receptor 2
TLR4: Toll-like receptor 4
TRPV4: Transient receptor potential vanilloid 4
WNK: With no lysine kinase
WT: Wild type
ZO-1: Zona-occludens 1
